# Inter-Annual Variations of Methane Emission from an Open Fen on the Qinghai-Tibetan Plateau: A Three-Year Study

**DOI:** 10.1371/journal.pone.0053878

**Published:** 2013-01-14

**Authors:** Huai Chen, Ning Wu, Yanfen Wang, Dan Zhu, Qiu’an Zhu, Gang Yang, Yongheng Gao, Xiuqin Fang, Xu Wang, Changhui Peng

**Affiliations:** 1 Chengdu Institute of Biology, Chinese Academy of Sciences, Chengdu, China; 2 Lab for Ecological forecasting and global change, College of Forestry, Northwest A&F University, Yangling, China; 3 University of Chinese Academy of Sciences, Beijing, China; 4 Institute of Mountain Hazards and Environment, Chinese Academy of Sciences, Chengdu, China; 5 Institut des Sciences de l’Environnement, Université du Québec à Montréal (UQAM), Montréal, Canada; 6 Research Institute of Tropical Forestry, Chinese Academy of Forestry, Guangzhou, China; The Ohio State University, United States of America

## Abstract

The study aimed to understand the inter-annual variations of methane (CH_4_) emissions from an open fen on the Qinghai-Tibetan Plateau (QTP) from 2005 to 2007. The weighted mean CH_4_ emission rate was 8.37±11.32 mg CH_4_ m^−2 ^h^−1^ during the summers from 2005 to 2007, falling in the range of CH_4_ fluxes reported by other studies, with significant inter-annual and spatial variations. The CH_4_ emissions of the year of 2006 (2.11±3.48 mg CH_4_ m^−2 ^h^−1^) were 82% lower than the mean value of the years 2005 and 2007 (13.91±17.80 mg CH_4_ m^−2 ^h^−1^ and 9.44±14.32 mg CH_4_ m^−2 ^h^−1^, respectively), responding to the inter-annual changes of standing water depths during the growing season of the three years. Significant drawdown of standing water depth is believed to cause such significant reduction in CH_4_ emissions from wetlands in the year 2006, probably through changing the methanogen composition and decreasing its community size as well as activating methanotrophs to enhance CH_4_ oxidation. Our results are helpful to understand the inter-annual variations of CH_4_ emission and provide a more reasonable regional budget of CH_4_ emission from wetlands on the QTP and even for world-wide natural wetlands under climate change.

## Introduction

Methane (CH_4_) is an important greenhouse gas, about 25 times more powerful in warming the atmosphere than carbon dioxide (CO_2_) for the time horizon of 100 years [1]. In particular, CH_4_ emissions have a larger impact on the climate than what was claimed in current carbon-trading schemes or in the Kyoto Protocol, which modified its radiative forcing as +0.48 W m^−2^ [2]. Given its atmospheric concentration, CH_4_ is regarded as an important greenhouse gas only second to CO_2_.

Due to the prevalence of waterlogged and anoxic conditions, wetlands are the largest natural source for atmospheric CH_4_ emission, about 148 Tg CH_4_ yr^−1^ (1 Tg = 10^12^ g) from natural wetlands [1], [3], contributing over 25% of the global CH_4_ emission to the atmosphere [4]. Moreover, wetlands represent not only one of the most important sources for methane emission, but also the most uncertain one. Such uncertainty arises primarily from the large spatiotemporal variation that occurs in different scales and the limited data of specific wetlands [1], [5]. Therefore, we need to fill into place the jigsaw pieces of data on specific wetlands from different regions, if we want to get the whole picture of CH_4_ emission from wetlands.

The Qinghai-Tibetan Plateau (QTP) is the largest and highest plateau in the world with an area of 2.5 million square kilometers. There are many lakes and wetlands on the plateau, with about 50% of wetlands and 51% of lakes of China unevenly distributed here [6]. On the eastern edge of QTP, there is the largest highland wetland in the world, Zoige alpine wetlands [7], which is, for its high altitude, a very important and sensitive area for climatic change [8], as well as hotspots for biodiversity in the world [9]. Though there are several studies about CH_4_ emission from wetlands on the plateau [10], [11], [12], [13], [14], [15], [16], these studies only discussed short-term variations of CH_4_ emission, not including inter-annual variation of CH_4_ emission and their determinants.

Wetlands on the QTP are sensitive to climate change and the plateau has experienced abrupt climate change [17]. In the past decades, trends of precipitation showed an overall slight increase with high inter-annual variations at the whole-plateau scale [18], [19]. This is also true for our study area. During the past 50 years, we observed a slight increase trend in the annual precipitation with high inter-annual variations. During our growing season measurements from 2005 to 2007, we encountered a dry year (2006) compared with the annual precipitation average during the period from 1957 to 2007. Moreover, in our study the chosen open fen was usually seasonally flooded, thus having obvious seasonal and inter-annual dynamics of standing water depths. This made an opportunity for us to test if CH_4_ emissions were significantly variable annually and if standing water depths were the dominant factor on inter-annual variations of CH_4_ emissions.

## Materials and Methods

### Ethics Statement

Our field studies were approved by Bureau of National Nature Reserve of Zoige Wetland. The study was observational, involving no cruelty to animals, no damage to habitats and no harm to endangered plants, and thus no review from the ethnic committee was required in China. All the work was carried out under the Wildlife Protection Law of the People's Republic of China.

### Site Description

**Figure 1:**
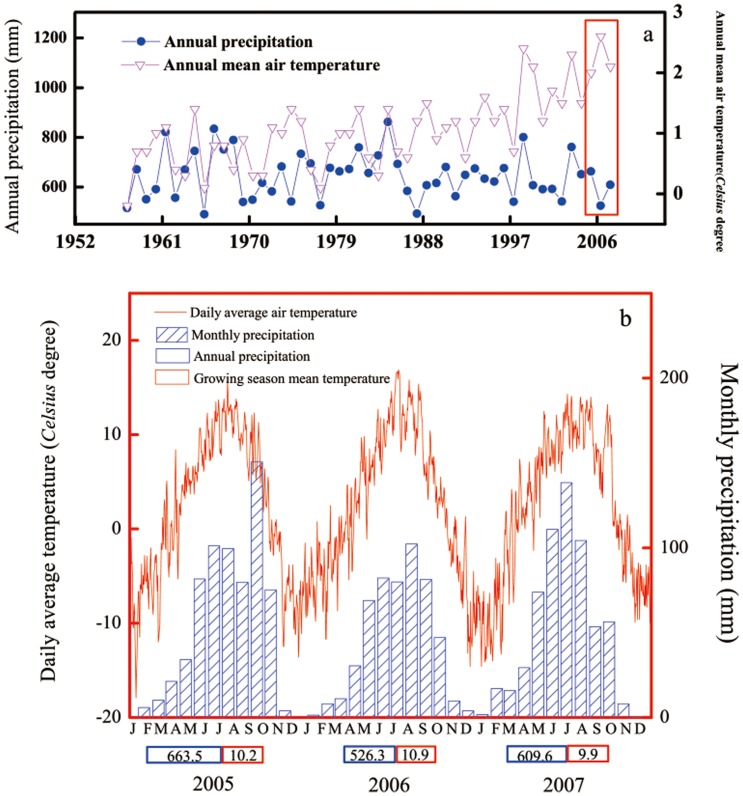
Weather conditions of Zoige. a. Annual air temperature and precipitation from 1957 to 2007 of Zoige County; b. Daily air temperature and precipitation from 2005 to 2007 in the study area.

The investigations were carried out in an alpine wetland of National Nature Reserve of Zoige Wetland (33°56′N,102°52′E, 3430 m a.s.l.), located on the northeast edge of the QTP. Zoige wetlands is on the Ramsar List of Wetlands of International Importance (2008), with ubiquitous alpine wetlands on the plateau formed during the Early Holocene (9355±115 BP) [20]. The region is characterized by cold Qinghai-Tibetan climatic conditions with average annual precipitation 645±92 mm and temperature 274.21±273.75 K from 1957 to 2007 (Fig. 1a).

A typical open fen was chosen in this study, which is about 28% of Zoige wetlands, covering an area of 7.08×10^5^ hm^2^ [7]. The fen is consisted of three stands, including *Kobresia tibetica* on the hummock (covering about 40% of the whole site), which is almost never flooded, emergent *Carex muliensis* and *Eleocharis valleculosa* stands in the hollow (covering about 25% and 35%, respectively), which are usually flooded with some sporadically drainages. Due to warming and hydrological dynamics, this fen is usually confronted with water table drawdowns in the mid-summer, especially for dry years.

### Weather and Soil Physical Characteristics

Local weather data were obtained from China Meteorological Data Sharing Service System (http://www.cma.gov.cn/2011qxfw/2011qsjgx/index.htm) from 1957 to 2007. During the monthly measurement of methane flux, air temperatures were also recorded.

Redox potentials and temperatures (soil and water) were taken with a portable digital meter (EcoScan pH6, Eutech Instruments Pte Ltd, Singapore). Water temperatures, ground surface temperatures and soil temperatures at the depth of 5 cm and 10 cm were manually recorded for each of the 18 plots. Standing water depths in the growing season were recorded with a ruler.

### Sampling Plots Establishment and CH_4_ Flux Measurement

Eighteen plots in the study site were established for the consecutive three growing seasons (July to September) from 2005 to 2007. Among the 18 plots, six were for *K. tibetica* stand, six for *C. muliensis* stand and six for *E. valleculosa* stand.

In the three years, we took monthly measurements from July to September. The CH_4_ emission was measured with vented static chambers [21], [22]. The chambers (30 cm in diameter, 50 cm in height) were made of cylindrical polyvinyl chloride (PVC) pipe. Details about the chambers were described in reference [12].

Four air samples from each chamber were taken at 10-minute intervals over a 30 minute period after enclosure, stored in 5 ml air-tight vacuumed vials. The CH_4_ concentration was determined by a gas chromatography (PE Clarus 500, PerkinElmer, Inc., USA), equipped with a FID (flame ionization detector), operating at 350°C and a 2 m Porapak 80–100 Q Column. The column oven temperature was 35°C and the carrier gas was N_2_ with a flow rate of 30 cm^3^ min^–1^.

The flux J of CH_4_ was calculated as:
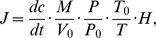
Where 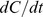 is the rate of concentration change; *M* is the molar mass of CH_4_; *P* is the atmosphere pressure of the sampling site; *T* is the absolute temperature of the sampling time; *V_0_, P_0_, T_0_* is the molar volume (22.4 L mol^−1^), atmosphere pressure (101.325 kPa), and absolute temperature (273.15 K), respectively, under the standard condition; *H* is the chamber height over the water surface.

### Calculation and Statistical Analysis

Mean CH_4_ emission, surface and soil temperature, Eh, and standing water depth for each stand type were calculated by averaging the replicates for each sampling date. A full general linear model in which stand and year were treated as fixed factors was used to compare the differences of environmental factors and CH_4_ emission in the three summers, and to assess the significance of the impacts of stand, year, and the combined effect of the two on CH_4_ emission and environmental factors. Multiple analysis of variance (MANOVA) was used to compare averages of CH_4_ emission for each stand of each sampling date and averages of CH_4_ emission for all stands in each year. The CH_4_ emissions were related to environmental variables by Pearson correlation analysis in each year. The effect of a certain variable was considered statistically significant for *P<*0.05. The above analyses were performed with the SPSS 11.5 for Windows.

## Results

### Variation in Air Temperature, Precipitation and Standing Water Depths

From the year 1957 to 2007, our study area showed a very obvious warming trend and a slight drying trend with significant inter-annual variations (*P*<0.01, Fig. 1a). During the past five decades, the average annual precipitation was 645±92 mm and the annual mean daily temperature was 1.06±0.6°C. For the experiment years (2005 through 2007) the annual mean precipitation and air temperature were 599 mm and 2.2°C (Fig. 1b). For each of the three years, the warmest month was July and the coldest month January. Also in all the three years more than 65% precipitation was distributed in the growing season (from June to September), about 431.1 mm in 2005, 354.7 mm in 2006 and 407.2 mm in 2007, with significantly less rainfall in 2006 than that in 2005 and 2007 (*P*<0.05). However, the growing season mean temperature was not significantly different among the three years (10.2°C in 2005, 10.9°C in 2006 and 9.9°C in 2007). During the three years, the lowest annual precipitation (526.3 mm) and the warmest mean daily air temperature (2.6°C) were recorded in 2006, a significantly drier and warmer year based on the annual averages of 1957 through 2007 (*P*<0.01).

**Figure 2:**
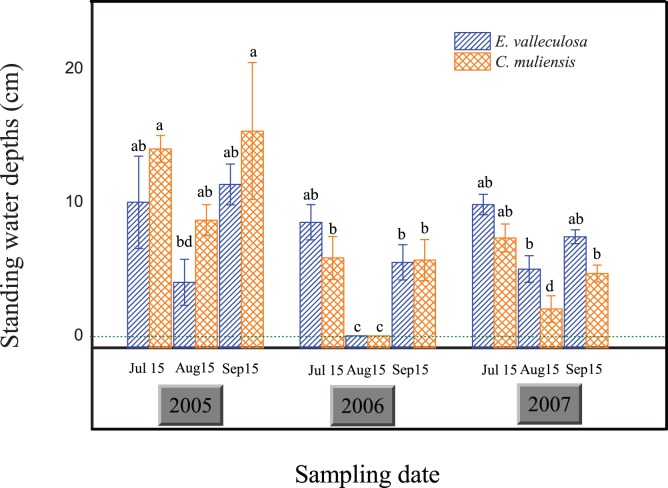
Standing water depths of the hollow stands during the growing seasons from 2005 to 2007. Different letters indicate significant difference (*P*<0.05).

During the summers of 2005 to 2007, standing water depths of the hollow stands (*C. muliensis* and *E. valleculosa*) varied markedly (Fig. 2). In the never-flooded hummock (*K. tibetica* stand), since water table was the height of hummocks from the surface of the standing water, it also varied greatly due to the dynamics of the standing water depths. Among the three stands, there were significant variations of standing water depths during the three summers (Table 1). However, standing water depths showed no significant difference between *C. muliensis* (6.8±3.7 cm) and *E. valleculos* stands (7.1±5.1 cm) except for that in July 2007. Moreover, the standing water depths of 2005 (10.6±4.5 cm) were significantly higher than that of 2006 (4.3±3.4 cm) and 2007 (6.0±2.7 cm), while there was no significant difference between the latter two.

**Table 1:** Significance of impacts of year, stand types and their combined effect on CH_4_ emission and environmental factors in growing season.

	Year	Stand types	The combined effect of year and stand
CH_4_ emission	**	**	*
Surface temperature	**	ns	ns
5 cm temperature	**	ns	ns
10 cm soil temperature	**	ns	ns
The standing water depth	*	**	**

* Significant impact P<0.05;

** highly significant impact, P<0.01; ns, no significant impact.

### CH_4_ fluxes from the Three Stands

**Figure 3:**
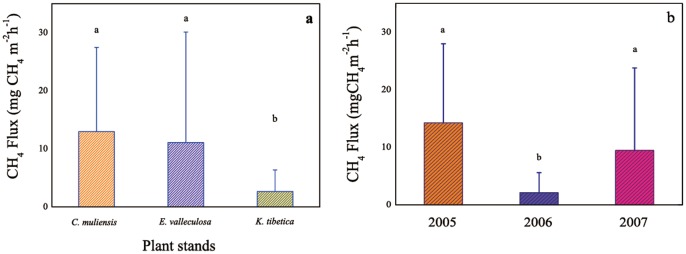
Spatiotemporal variation of CH_4_ fluxes. a. Mean CH_4_ fluxes in different stands during the growing seasons; b. Inter-annual variation of CH_4_ emission from the open fen of 2005 to 2007. Different letters indicate significant difference for each panel (*P*<0.05).

**Figure 4:**
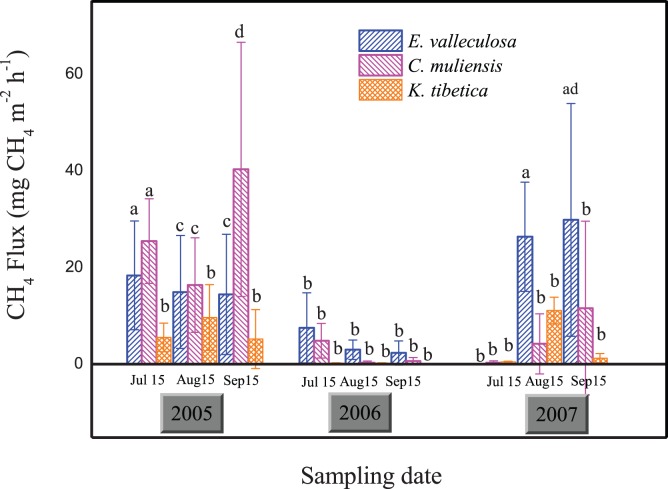
Seasonal variation of CH_4_ emission from the three plant stands from 2005 to 2007. Different letters indicate significant difference (*P*<0.05).

We found different stands had different CH_4_ emission during the study period (Fig. 3). The CH_4_ emission (mean ± SD) from the open fen was about 8.68±14.33 mg CH_4_ m^−2 ^h^−1^. The *C. muliensis* stand emitted CH_4_ at the highest rate, about 12.97±14.50 mg CH_4_ m^−2 ^h^−1^. The *K. tibetica* stand emitted CH_4_ at the lowest rate, about 2.65±3.74 mg CH_4_ m^−2 ^h^−1^, and the *E. valleculosa* stand emitted CH_4_ at an intermediate emission rate about 11.09±19.04 mg CH_4_ m^−2 ^h^−1^. Comparing the three-year means of each stand, we also found that CH_4_ emission from *C. muliensis* and *E. valleculosa* stands was significantly higher than that from *K. tibetica* stand, with no significant difference between the former two (Fig. 3a). However, this trend was not the same for each year. For example, CH_4_ emission from *C. muliensis* and *E. valleculosa* stands was significantly higher than that from *K. tibetica* stand in the years 2005 and 2007, while there was no significant difference among the three stands in 2006 (Fig. 4).

### Seasonal and Inter-annual Variations of CH_4_ Fluxes

In this study, we found CH_4_ emissions of the year of 2006 (2.11±3.48 mg CH_4_ m^−2 ^h^−1^) were significantly lower than that of 2005 and 2007 (13.91±17.80 mg CH_4_ m^−2 ^h^−1^ and 9.44±14.32 mg CH_4_ m^−2 ^h^−1^, respectively), with no significant differences between 2005 and 2007 for all the three stands (Fig. 3b). The trend varied with stands. We observed significant inter-annual and seasonal variations of CH fluxes in *C. muliensis* and *E. valleculosa* stands of 2005 and 2007 (Table 1). There was no significant inter-annual or seasonal variation of CH_4_ emission in *K. tibetica* stand, with the emission rate markedly higher in 2005 and 2006 than in 2007 (Table 1).

### Key Factors Controlling CH_4_ Fluxes

There were many factors influencing CH_4_ fluxes, including soil temperatures, soil redox potentials, standing water depth and the community height, *etc* (Table 2). However, in different years, they influenced CH_4_ fluxes differently. In the year of 2005, we found CH_4_ fluxes significantly related to surface temperature, soil redox potentials (at 5 cm, 10 cm and 15 cm soil depths), standing water depths and the plant community height. In the year of 2006, CH_4_ fluxes were related to soil redox potentials (at 10 cm and 15 cm soil depths), standing water depths and the plant community. In the year of 2007, CH_4_ fluxes were just related to soil temperatures (at 5 cm and 10 cm soil depths) and plant community heights. In different stands, the relations were also different during the three-year period. In the *E. valleculosa* stand, we found surface temperatures, soil temperatures (at 5 cm and 10 cm soil depths) and plant community heights significantly correlated to CH_4_ fluxes. In the *C. muliensis* stand, only standing water depths and plant community heights were significantly correlated with CH_4_ fluxes. In the *K. tibetica* stand, only soil temperatures (at 5 cm and 10 cm soil depths) and plant community heights were significantly correlated to CH_4_ fluxes.

**Table 2:** Significance of Pearson's rank correlations between CH_4_ emission and environmental factors.

	CH_4_ emission rates					
	2005	2006	2007	*E.valleculosa*	*C.muliensis*	*K.tibetica*
Surfacetemperature	**	ns	ns	*	ns	ns
5 cm soiltemperature	ns	ns	*	*	ns	**
10 cm soiltemperature	ns	ns	**	*	ns	*
5 cm soil Eh	**	ns	ns	ns	ns	nd
10 cm soil Eh	**	*	ns	ns	ns	nd
15 cm soil Eh	**	*	ns	ns	ns	nd
The Standingwater depth	**	**	ns	ns	**	nd

ns indicates the correlation is not significant.

* indicates the correlation is significant (*P*<0.05);

** indicates the correlation is highly significant (*P*<0.01); nd means no data.

## Discussion

### Comparisons with Other Studies on the QTP

The weighted mean CH_4_ emission rate was about 8.37±11.32 mg CH_4_ m^−2 ^h^−1^ during the growing seasons from 2005 to 2007 with great inter-annual and spatial variations, falling in the range of CH_4_ fluxes during the growing seasons reported by other studies (summarized in Table 3). CH_4_ fluxes from wetlands show a significant spatial variations on the plateau [23], while their temporal variations are similar during the growing seasons [12], [13], [15]. With a large total area (*ca.* 1.33×10^5^ km^2^) of wetlands on the QTP, the entire plateau is a source of CH_4_ in summer with high spatiotemporal variations [23]. Based on the distribution of wetlands, representative CH_4_ fluxes, and number of thaw days, a preliminary estimate is *ca.*0.7–0.9 Tg CH_4_ yr^−1^ emitting from wetlands on the plateau [15]. However, for a more reasonable estimate, we need a greater amount of observational field data at various temporal and spatial scales, hopefully through establishing a high-proficiency and long-term monitoring network in the future.

**Table 3:** Comparison with other studies about methane flux from wetlands on the Qinghai-Tibetan Plateau reported with the static chamber method.

Location	Vegetation	Methane flux (mg CH_4_m^-2 ^h^-1^ )	Study period		Reference	
In Zoige County	*K. tibetica*	5.49±5.29	Jun. to Sept.2005 to 2007		This study	
(33°56′N,102°52′E, a.s.l. 3430 m)	*C. muliensis*	17.78±17.50				
	*E. valleculosa*	13.35±12.27				
In the Huashixia Region	*Carex meadow*	0.41±0.79	Apr. to Sept.1997		[15]	
(35°39′N, 98°48′E, a.s.l. 4300–4500 m)	*Caltha scaposa*	±0.28				
	*Hippuris vulgaris*	1.46±2.30				
	*C. atrofusaskr*	3.00±4.25				
In Hongyuan County	*C. muliensis*	2.87 (0.51-8.20)	May to Sept.2001		[16]	
(32°47′N, 102°32′E, a.s.l. 3470 m)	*C. meyeriana*	4.51 (0.36-10.04)				
In the Lanhaizhi wetland	*Potamogeton*	1.38	Jul. to Sept.2002	[13]		
(37°29′N, 101°12′E, a.s.l. 3250 m)	*Hippuris*	8.92				
	*Scirpus*	4.57				
	*Carex*	8.19				
In Haibei	*Carex meadow*	0.80–1.41	Jun. to Sept.2003			
(37°37′N, 101°19′E, a.s.l. 3280 m)	*Carex and Hippuris*	2.91–16.25				
In Zoige County (littoral wetlands)	*K. tibetica*	-0.1–26.3	Jun. to Aug.2005 and 2006		[10]	
(33°56′N,102°52′E, a.s.l. 3430 m)	*C. muliensis*	-0.1–21.8				
	Non-vegetated	0.1–3.8				
	*H. vulgaris*	0.2–22.9				
	*P. amphibium*	0.4–40.4				
	*G. maxima*	12–90				

### Role of Standing Water Depth in the Inter-annual Variations of CH_4_ Fluxes and its Implications for the Future

This study found that significant inter-annual and spatial variations of CH_4_ fluxes from the open fen (Table 1). In our previous studies and other related ones, standing water depths were regarded as the dominant factors controlling seasonal and spatial variations of CH_4_ fluxes [12], [24], [25]. Consistent with results from two Michigan peatlands [26], standing water tables were also the dominant factor influencing inter-annual variation of CH_4_ emissions, with their correlation relatively small but significant (r = 0.17, *P*<0.05). Furthermore, we noted that in the year 2006 with the thorough drainage (totally drying without standing water in the hollow) in July, CH_4_ fluxes from the open fen was 82% lower than the mean value of the years 2005 and 2007 (Fig. 3b). Similar to our study, a climate-induced drainage in summer was found to limit CH_4_ emission from newly created marshes [27]. In rice paddies, mid-season drainages also greatly reduced CH_4_ emissions [28], [29]. Consistent results were also found in stimulated drying experiments in wetlands [30], [31], [32], [33], [34]. Significant drawdown of water table position or standing water depth is believed to explain such significant reduction in CH_4_ emissions from the open fen in 2006 [34], [35], because more aerobic conditions enhanced CH_4_ oxidation and suppressed CH_4_ production and emission during the drawdown period [36], [37]. Also water table drawdown could have altered the structure of soil microbial communities related to methanogenesis and methanotrophs, which in turn limited CH_4_ production and enhanced CH_4_ oxidation [38]. In a very recent paper for the same studying site, we found methanogens community composition changed after the significant water table drawdown in 2006 and the community size was 10-time smaller in 2006 than that of 2007 [39]. The present study also showed that CH_4_ emission depended more on standing water depth in the relative dry year 2006 than 2005 and 2007, which were both relatively humid (Table 2). Furthermore, the water table drawdown in 2006 was also found to change the spatial patterns of CH_4_ emission among the three stands. In 2006, there were no significant variations of CH_4_ emission among the three stands; while in 2005 and 2007, CH_4_ emission was significantly higher in the flooded *C. muliensis* and *E. valleculosa* stands than in the dry *K. tibetica* stand [12]. This is partly because all three stands were dry in this year, and there was no significant variation of standing water depths among the hollow stands through significant drawdown of water tables (Fig. 2). For better understanding of CH_4_ dynamics of wetlands after significant water regime shifts, changes in soil microbial communities, vegetation cover and enzyme activities are the research priorities.

Although there is a slight wetting trend on the QTP with high inter-annual variations [19], [40], together with warming, wetlands on the plateau experienced great inter-annual changes and degraded during the last several decades [7], [41]. Such inter-annual dynamics in wetland area was believed to be the dominant cause of inter-annual variations in regional CH_4_ emissions from wetlands [42]. Moreover, inter-annual variations not only resulted in changes of wetland areas at large scales, but also in changes of water tables of specific wetlands. Therefore, inter-annual changes of water tables or hydrological processes should be another determinant of inter-annual variations of CH_4_ emissions from wetlands. In our study, the water table drawdown in 2006 may not only lead to reduction in wetland area but also to decreased CH_4_ emission rate from previous emission “hotspots” of CH_4_ [12], which could make the inter-annual variation of CH_4_ emission greater. Due to wetland degradation, the CH_4_ source strength of the entire QTP wetlands has declined during the past 50 years with highly inter-annual variations responding to highly inter-annual hydrological regime of wetlands under climate change [19]. As an example, sporadic drought events like that in 2006 may further decrease CH_4_ emission rate from typical wetlands on the plateau, making CH_4_ source of the entire plateau wetlands smaller and more variable. The results of the present research are meaningful to understanding the inter-annual variations of CH_4_ emission and getting a more reasonable regional budget of CH_4_ emission from wetlands on the QTP and even for world-wide natural wetlands under climate change.
